# Plant Viruses, Viroids and Phytoplasmas: Insight into Evolutionary, Pathogenicity, and Epidemiology Studies

**DOI:** 10.3390/plants14040551

**Published:** 2025-02-11

**Authors:** Luca Ferretti, Anna Taglienti

**Affiliations:** Council for Agricultural Research and Economics (CREA), Research Centre for Plant Protection and Certification, Via C.G. Bertero, 22, 00156 Rome, Italy

Plant viruses, viroids, and phytoplasmas are systemic, unculturable pathogens that are increasingly endangering sustainable food security and environmental safety worldwide. Though many viruses do not cause severe disease in plants, instead producing symptomless or mild syndromes with limited impact on growth and yield, several of them trigger physiological disorders resulting in significant losses [1], affecting both the yield and quality of agricultural crops. Economically important viruses infect agriculturally relevant staple crops or plants with high added value (e.g., cassava, potato, citrus, grapes). Such damage accounts for USD 30 billion losses in agricultural crops every year [2]. Moreover, modern cropping techniques such as monocropping, high-density cropping, and uniform genetics agriculture practices, together with global trade exchanges and climate change issues, pave the way for the emergence of new pathogens.

The scenario is further worsened by viral pathogens because no treatments are available to cure the associated diseases in the field, and the only available strategies rely on prevention (i.e., the use of certified virus-free plant propagation material, the control of insect vectors, or the use of resistant varieties).

In view of the above, the plant health field relies heavily on the genomic characterisation of viral pathogens for many applications. Firstly, the knowledge of genomic sequences allows for the development of molecular diagnostic tests, which are crucial for the prompt recognition of outbreaks, allowing proper control measures to be set up and the impact to be reduced. Genomic characterisation is also essential for taxonomic classification, which is now largely based on the molecular features of genomes and can involve highly complex procedures, especially in the case of phytoplasmas. Finally, phylogenetic analysis is based on the genomic traits of viruses and is a major tool for unravelling genetic diversity and the organisation of plant virus populations for evolutionary, pathogenicity, and epidemiology studies.

The aim of this Special Issue was to gather studies focused on a broad variety of topics regarding the identification and molecular characterisation of plant viruses, viroids, and phytoplasmas. Out of the eight published papers, six focused on viruses and two on phytoplasmas. The papers regarding viruses addressed two main research categories that focus on basic molecular and biological characterisation, respectively.

The first category consists of three papers. In the first one, a molecular identification of viruses infecting hop in Italy was provided [3], and phylogenetical analysis was performed using the genomic sequences obtained.

Hop (*Humulus lupulus* L.) is a minor ingredient in beer; its female flowers are used in the brewing industry to impart bitterness, aroma, and flavour. The presence of viral infections in the plant is reported to severely alter the chemical composition of the hop cones; in Italy, the presence of the *Carlavirus* genus in hop was already known, but no information was yet available on species identification. This work aimed to fill this gap by identifying the presence of different carlaviruses and explore the molecular features and variability of Italian isolates.

Hop latent virus (HpLV) was found to be the most widespread species, with 96% incidence, while a small number of samples was infected by hop mosaic virus (HpMV, 6%) or American hop latent virus (AHLV, 4%). Mixed infections were also not very common (6%).

Phylogenetic analysis performed on RT-PCR amplicons targeting the ORF5s, encoding the coat protein (CP), showed that the HpLV Italian isolates were grouped into two distinct and highly separated clusters. HpMV and AHLV isolates were characterised by distinct features compared to the identified clusters, and mainly grouped together in a separate branch of the respective phylogenetic trees.

Moreover, HTS RNA-seq analyses enabled complete genomes of the three virus species in the selected samples to be assembled, and a phylogenetic tree was also constructed with these genomes and reference genomes of carlaviruses in GenBank, thus positioning the Italian isolates in the tree and allowing their phylogenetical relatedness to be analysed. Such improvements in the knowledge of the molecular, phylogenetic, and epidemiological features of hop viruses in Italy meet the aims of this Special Issue and may pave the way for the efficient management of the diseases with the most impact. Moreover, better knowledge may help in the development of a certification scheme for sanitarily controlled hop germplasm.

The second paper falling into the basic molecular characterisation category reported the near-complete genomic sequencing of a Czech isolate of Erysimum latent virus (ELV) using contemporary high-throughput methods [4]. A comparison between this isolate and a German isolate (ELV-De) in GenBank, whose sequence was obtained previously using the Sanger method, confirmed the correctness of the deposited ELV-De genome, its similarity to the Czech isolate, and the concurrent considerable distance of both isolates from other tymoviruses. Nonetheless, the analysis of the MP/RdRp protein contributes to understanding the role of the MP gene in the spread and symptoms of tymoviruses. Overall, these results highlight the close relationship between sequence and functional features, which are even more important when HTS is exploited to the point of single-nucleotide polymorphism detection.

The last paper reporting molecular characterisation studies focused on the isolation of a comovirus in wild asymptomatic *Brassica hirta* (white mustard) plants harbouring a dense population of *Brevicoryne brassicae* aphids [5]. The study explored the epidemiology and pathogenicity of this comovirus isolate through biological and molecular tools, highlighting its ability to cause ringspot symptoms solely in *B. rapa* var. perviridis plants despite its quite wide host range. Due to the homology with *Apis mellifera*-associated comovirus and the efficiency of mechanical inoculation, the virus was hypothesised to be transmitted by non-host vectors via mechanical adherence through bee colonies. In fact, HTS analysis enabled the retrieval of the full genome of the newly isolated virus, and the low virus load was in accordance with a non-replicating virus in a non-host vector where the viral particles externally adhered to the bees and were transmitted by passive adherence via floral visitation. The paper ultimately suggests a possible contribution of managed and wild pollinators to the long-term adaptation of plants to a seed-borne latent comovirus. Such results shed new light on the epidemiology of comoviruses, to date reported as seed-transmitted viruses; an accurate knowledge of the main routes of transmission is of capital importance in the management and control of virus diseases in plants.

Three papers were included in the second category, identified as biological characterisation. The first one reported the results of a study aimed at providing a basic understanding of the mechanism of infection of *Brassica juncea* plant (leaf mustard) by cucumber mosaic virus (CMV), an economically important virus distributed worldwide but rarely investigated in this crop [6]. The results highlighted the role of CMV RNA2 as the main determinant, and the specific amino acids in the N-terminal region of 2a protein as key players in the systemic infection of CMV in *B. juncea* and the encoded proteins involved. From this perspective, the paper represents an important contribution useful for clarifying and better understanding the complex mechanism underlying the systemic infection of plant hosts by viruses.

The second paper in this category provided the biological characterisation of a new isolate of tomato mottle mosaic virus (ToMMV) causing a newly emerging disease in tomato plants in India. This disease is characterised by distinctive shoestring symptoms on the leaves and the development of unripe, small, and hard fruit, resulting in substantial reductions in fruit yield and quality [7]. By means of transmission electron microscopy (TEM) and molecular analyses, an isolate of the tomato mottle mosaic virus (ToMMV) was identified in the affected plants and its causal relationship with the disease was confirmed through biological testing. When the partially purified virus was mechanically inoculated into tomato cv. Pusa Ruby plants, it reproduced the characteristic shoestring symptoms. Host range studies were also carried out, demonstrating that the shoestring isolate of ToMMV (ToMMV-Tss) can infect several solanaceous species, while cucurbitaceous hosts remain unaffected. In the paper authors also provided evidence that ToMMV-Tss isolate can induce similar shoestring symptoms in most of the major commercial tomato varieties when inoculated under controlled experimental conditions in the glasshouse, indicating its aggressive nature. Moreover, the virus was found to be seed-transmissible, even if only a small percentage of seedlings from infected plants displayed symptoms. In line with the aims and contents of this Special Issue, the paper provides new and relevant data on the epidemiology and pathogenicity of this new ToMMV isolate, underscoring its potentially significant impact on tomato production and the need for reliable diagnostic tools and effective management strategies to curb its spread and mitigate the impact of this virus on commercial tomato cultivation.

The third paper involving biological characterisation was an interesting study on the labelling and tracking of PMMoV CP in vivo to monitor its systemic spread at spatial and temporal scale during the viral cycle [8]. The cargo protein was fused to the fluorescent tag using the ribosomal skip principle: the insertion of a 2A 18-mer peptide derived from foot-and-mouth disease virus between the CP and GFP, which modifies the activity of the ribosome to promote the hydrolysis of the peptidyl(2A)-tRNA(Gly) ester linkage, hence releasing the polypeptide from the translational complex. This process produces a ribosomal “skip” from one codon to the next without the formation of a peptide bond, allowing the formation of cargo-2A-CP, free cargo, and free CP. The vector was delivered to *Nicotiana benthamiana* through electroporated *Agrobacterium tumefaciens* and proved to be infectious. Western blot detected expected bands at the molecular weights of free GFP and fused protein, while quantitative PCR assays also indicated the virus presence. The integrity of the constructed vectors was further demonstrated by TEM observations of extracted and purified virions, which shared the typical rod-like structure, length, and diameter compatible with the tobamovirus particles. The TEM observations also showed that virus particles produced by the vector incorporate GFP; confocal microscopy confirmed that tracking GFP allowed the dynamic changes of the CP to be followed, and that virions or virion aggregation can form fluorescent plaques. The system based on the cargo protein fused to the fluorescent tag proved to be a valuable tool for investigating the spatial and temporal dynamics of PMMoV CP during viral infection. The published paper offers a basic methodological approach that is potentially useful for studying the complex infection process of the host plant and the movement of the viral particles in the plant tissues, thus also being useful in the development of possible control strategies.

The two papers focused on phytoplasmas addressed two different research fields. The first paper reported the results of an epidemiological investigation carried out in Serbia to better understand the epidemiological pathways of “*Candidatus* Phytoplasma solani” (CaPsol) in relation to its occurrence in economically important crops [9]. The investigation was based on a multi-test study integrating field (sampling of naturally CaPsol-infected insect vectors from the planthopper family Cixiidae) and laboratory (experimental CaPsol transmission trials to economically important plants) work to ensure comprehensive results that reflect in-field events. From this perspective, the paper provides an effective methodological approach applicable to different plant–pathogen models for predicting possible epidemiological risk scenarios.

The second paper focusing on phytoplasmas addressed the critical issue of their detection using HTS technology, represented by the quality and amount of phytoplasma DNA isolated from the plant host DNA. This issue is a key factor for obtaining high-quality DNA sequencing data [10] considering that phytoplasma DNA is often much less abundant than the massive amount of host plant DNA, making isolation difficult. In addition, samples often contain DNA from smaller genomes that make the isolated DNA less suitable for downstream applications such as whole-genome sequencing. In response to these challenges, in this study, a novel, simple, and cost-effective alternative method for DNA isolation was developed to avoid the use of hazardous chloroform and to enable the selection of DNA fragments by size, facilitating the enrichment of microbial DNA. Despite the study mainly focusing on technical and methodological issues, apparently not fulfilling the aims of the Special Issue, it provides a robust framework for studying plant pathogens in complex plant models, resulting in wide and multidisciplinary scientific interest.
